# Digital medicine, on its way to being just plain medicine

**DOI:** 10.1038/s41746-017-0005-1

**Published:** 2018-01-15

**Authors:** Steven R. Steinhubl, Eric J. Topol

**Affiliations:** Scripps Translational Science Institute, 3344 North Torrey Pines Court, Suite 300, La Jolla, CA 92037 USA

**Keywords:** Public health, Translational research, Health care, Health sciences, Medical research

There are already nearly 30,000 peer-reviewed English-language scientific journals, producing an estimated 2.5 million articles a year.^1^ So why another, and why one focused specifically on digital medicine?

To answer that question, we need to begin by defining what “digital medicine” means: using digital tools to upgrade the practice of medicine to one that is high-definition and far more individualized. It encompasses our ability to digitize human beings using biosensors that track our complex physiologic systems, but also the means to process the vast data generated via algorithms, cloud computing, and artificial intelligence. It has the potential to democratize medicine, with smartphones as the hub, enabling each individual to generate their own real world data and being far more engaged with their health. Add to this new imaging tools, mobile device laboratory capabilities, end-to-end digital clinical trials, telemedicine, and one can see there is a remarkable array of transformative technology which lays the groundwork for a new form of healthcare.

As is obvious by its definition, the far-reaching scope of digital medicine straddles many and widely varied expertise. Computer scientists, healthcare providers, engineers, behavioral scientists, ethicists, clinical researchers, and epidemiologists are just some of the backgrounds necessary to move the field forward. But to truly accelerate the development of digital medicine solutions in health requires the collaborative and thoughtful interaction between individuals from several, if not most of these specialties. That is the primary goal of *npj Digital Medicine*: to serve as a cross-cutting resource for everyone interested in this area, fostering collaborations and accelerating its advancement.

Current systems of healthcare face multiple insurmountable challenges. Patients are not receiving the kind of care they want and need, caregivers are dissatisfied with their role, and in most countries, especially the United States, the cost of care is unsustainable. We are confident that the development of new systems of care that take full advantage of the many capabilities that digital innovations bring can address all of these major issues. Researchers too, can take advantage of these leading-edge technologies as they enable clinical research to break free of the confines of the academic medical center and be brought into the real world of participants’ lives. The continuous capture of multiple interconnected streams of data will allow for a much deeper refinement of our understanding and definition of most phenotypes, with the discovery of novel signals in these enormous data sets made possible only through the use of machine learning.

Our enthusiasm for the future of digital medicine is tempered by the recognition that presently too much of the publicized work in this field is characterized by irrational exuberance and excessive hype. Many technologies have yet to be formally studied in a clinical setting, and for those that have, too many began and ended with an under-powered pilot program. In addition, there are more than a few examples of digital “snake oil” with substantial uptake prior to their eventual discrediting.^2^ Both of these practices are barriers to advancing the field of digital medicine.

Our vision for *npj Digital Medicine* is to provide a reliable, evidence-based forum for all clinicians, researchers, and even patients, curious about how digital technologies can transform every aspect of health management and care. Being open source, as all medical research should be, allows for the broadest possible dissemination, which we will strongly encourage, including through advocating for the publication of preprints

And finally, quite paradoxically, we hope that *npj Digital Medicine* is so successful that in the coming years there will no longer be a need for this journal, or any journal specifically focused on digital medicine. Because if we are able to meet our primary goal of accelerating the advancement of digital medicine, then soon, we will just be calling it medicine. And there are already several excellent journals for that.
